# How yoga shapes the brain: a systematic review

**DOI:** 10.3389/fnins.2026.1718808

**Published:** 2026-04-13

**Authors:** Laura Stoelers, Samuel Arias-Sánchez, Paloma Domínguez, Isabel Martín-Monzón

**Affiliations:** 1 Faculty of Psychology, Campus Santiago Ramón y Cajal, University of Seville, Seville, Spain; 2 Department of Experimental Psychology, Faculty of Psychology, Campus Santiago Ramón y Cajal, University of Seville, Seville, Spain; 3 Wellness Neuroscience Group, Department of Experimental Psychology, Faculty of Psychology, Campus Santiago Ramón y Cajal, University of Seville, Seville, Spain

**Keywords:** brain structure, functional connectivity, neuroimaging, structural activity, yoga

## Abstract

Yoga is a mind–body practice that originated in India thousands of years ago, and which has extended throughout the world in recent years. As it becomes more popular, more studies are being conducted regarding its health benefits in multiple areas, including the human brain, where results have shown that it can reduce stress, modulate neurotransmitters, increase cerebral blood flow, and affect brain structure and function. This review aims to provide a synthesis of the current knowledge on the impact of yoga on human brain structure and function, through the selection and analysis of 23 international peer-reviewed neuroimaging studies with healthy participants. These studies were selected from 216 results on Web of Science, PubMed and PsycInfo after applying the inclusion and exclusion criteria. The final set of studies employed both neuroimaging and neurophysiological techniques, including MRI, fMRI, and EEG. The results show that yoga may exert multiple effects on the brain. However, the heterogeneity of results may be explained by differences in sample characteristics, study designs, and the lack of a consistent definition of yoga and its distinction from meditation. Finally, the limitations of the present review are discussed, along with recommendations for future research aimed at better understanding the neuropsychological health benefits of yoga.

## 1 Introduction

Yoga is a mind–body practice with historical roots in ancient Indian traditions (Nagendra, 2008; Satchidananda, 2012). While traditionally embedded within a broader philosophical system, contemporary yoga encompasses a range of practices that are increasingly studied for their effects on psychological and biological functioning (Gard et al., 2014a; Schmalzl et al., 2015; van Aalst et al., 2020). In the present review, yoga is considered primarily as a set of practices relevant to brain structure and function, rather than in its historical or philosophical dimensions (Afonso et al., 2021; Bakshi and Srivastava, 2024; Foxen and Kuberry, 2021; Davies et al., 2024).

Contemporary yoga includes diverse styles that differ in their relative emphasis on physical postures (*asana*), breath regulation (*pranayama*), meditation (*dhyana*), and sensory withdrawal (*pratyahara*) (Schmalzl et al., 2015; Cramer et al., 2016a). Some practices prioritize sustained postures combined with controlled breathing (e.g., Hatha-based approaches), whereas others focus predominantly on meditative components with minimal physical movement. More dynamic styles, such as Vinyasa- or Ashtanga-based practices, integrate continuous movement and breath coordination (Cramer et al., 2016a). These variations are relevant for neuroimaging research, as different components of yoga may differentially engage motor, attentional, interoceptive, and self-regulatory neural systems, thereby contributing to heterogeneity across study findings (Gard et al., 2014a; van Aalst et al., 2020).

Over recent decades, yoga practice has expanded globally and gained widespread popularity, particularly in Europe and North America, largely driven by its use for health-related purposes and as a complementary approach to psychological and medical treatments (Clarke et al., 2015; Cramer et al., 2016b; Zhang et al., 2021). Consequently, increasing research interest has focused on the potential benefits of yoga for physical health, emotional well-being, and cognitive functioning (Kinser et al., 2012; Gothe et al., 2019; Nourollahimoghadam et al., 2021; Bhargav et al., 2024; Gautam et al., 2024; Olex and Olex, 2018; Cramer et al., 2018).

**Figure 1:**
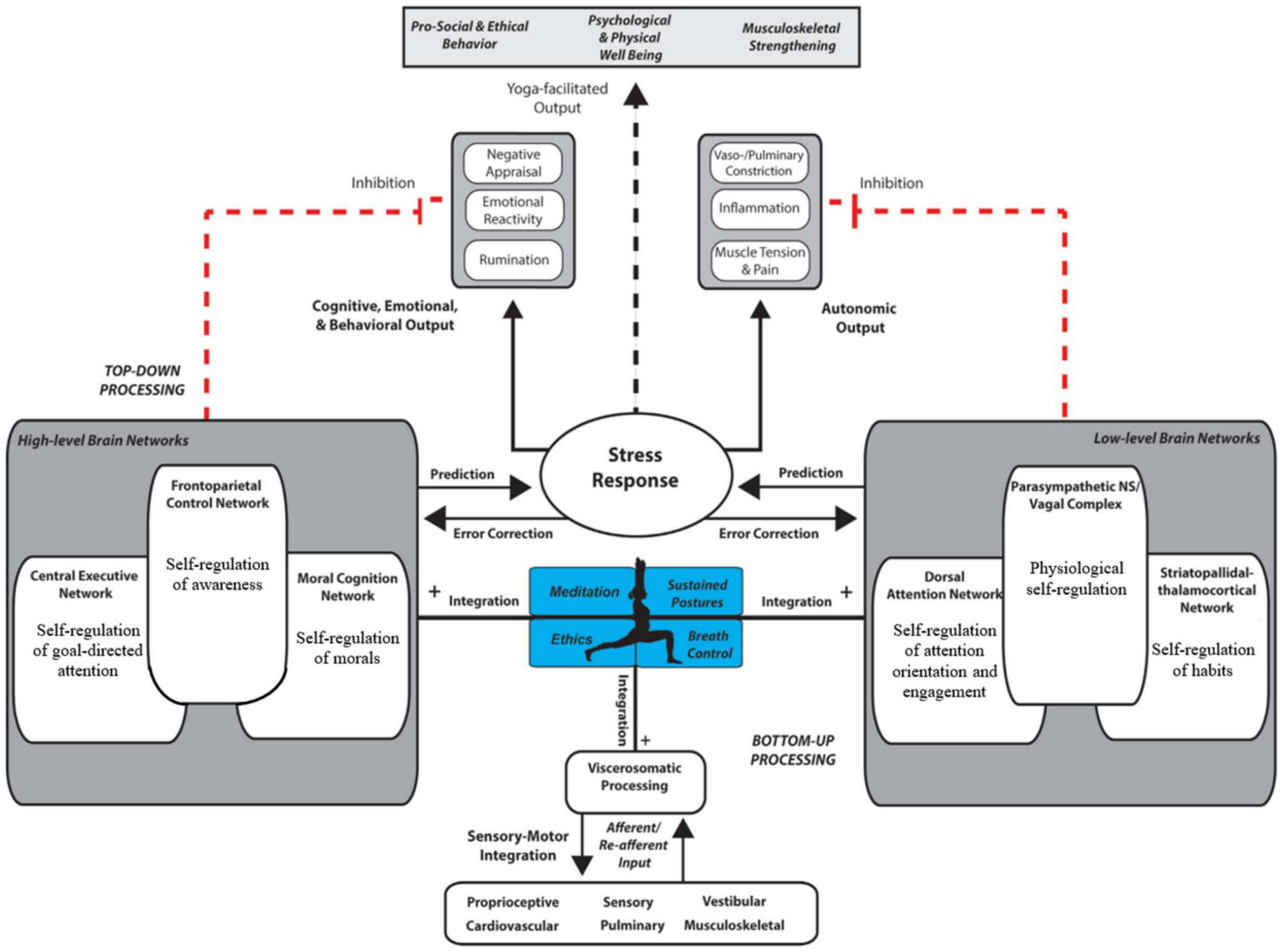
Top-down and bottom-up regulation through yoga practice. Modified from Gard et al. (2014a,b).

**Figure 2:**
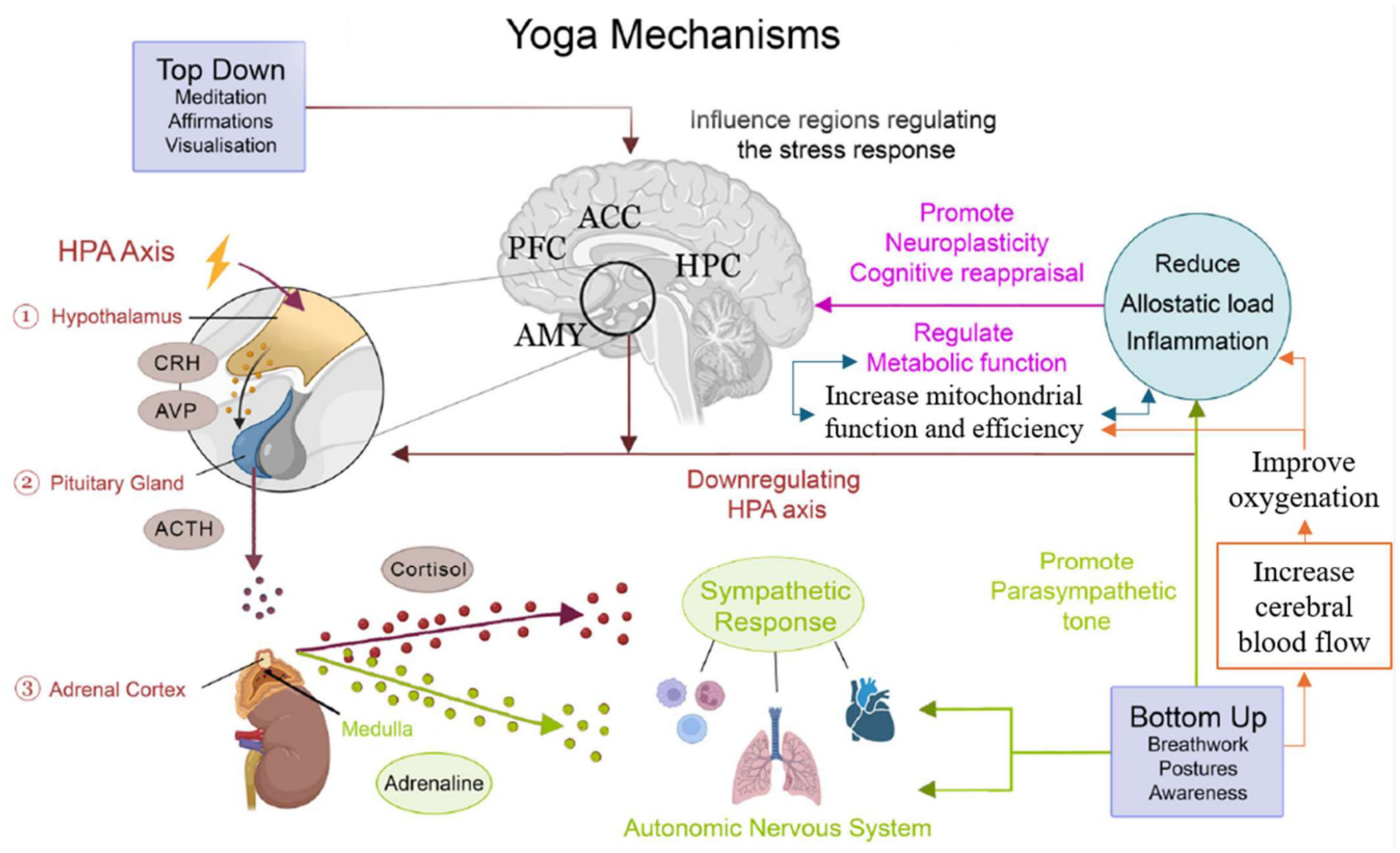
The interaction between the HPA pathway, cerebral blood flow and mitochondrial function. PFC, prefrontal cortex; ACC, anterior cingulate cortex; HPC, hippocampus; HRV, heart rate variability; AMY, amygdala, HPA axis, hypothallamic–pituitary–adrenal axis. Modified from Chiarpenello and Brodmann (2024).

From a neurobiological perspective, yoga has been proposed to influence brain function through multiple interacting pathways, including modulation of the autonomic nervous system, regulation of the hypothalamic–pituitary–adrenal (HPA) axis. It has also been associated with changes in neural networks involved in attention, emotion regulation, and self-referential processing (Taylor et al., 2010; Schmalzl et al., 2015; Aggarwal, 2020). Several theoretical frameworks have been proposed to explain these effects. Taylor et al. (2010) describe an executive homeostatic network (EHN), involving prefrontal, cingulate, and insular regions, which integrates cognitive, emotional, and physiological information to support self-regulation. Similarly, Gard et al. (2014a,b) propose a self-regulatory model of yoga emphasizing the interaction between top-down cognitive control processes and bottom-up physiological regulation across attentional, emotional, and autonomic domains. Importantly, these frameworks provide conceptual models for interpreting potential effects of yoga practice, but they do not constitute direct empirical evidence (see Figures 1, 2) (Davidson et al., 2003; Li et al., 2020; Rathore et al., 2022; Kip and Parr-Brownlie, 2023).

An increasing number of neuroimaging studies have investigated the effects of yoga on brain structure and function using techniques such as electroencephalography (EEG), structural magnetic resonance imaging (MRI), and functional MRI (fMRI) (Desai et al., 2015; Fox et al., 2014; Gothe et al., 2018; van Aalst et al., 2020). Structural and functional MRI studies have reported changes in brain regions involved in executive control, emotional regulation, interoceptive awareness, and default mode network (DMN) dynamics (Gard et al., 2014b; Villemure et al., 2015; Gothe et al., 2018; Tomasello et al., 2023; Konecki et al., 2016; Nayan Rishi et al., 2024).

EEG is a widely used technique to study the neural correlates of yoga and meditation, as it provides information about oscillatory brain activity associated with different cognitive and affective states (Bell and Cuevas, 2012; Kora et al., 2021). Previous research has shown that yoga and meditative practices are commonly associated with increases in alpha and theta activity, which have been linked to relaxation, internalized attention, and emotional regulation (Desai et al., 2015; Thomas et al., 2014; Martínez et al., 2020). In more experienced practitioners, changes in higher-frequency bands such as gamma have also been reported, potentially reflecting enhanced moment-to-moment awareness (Thomas et al., 2014; Martínez et al., 2020). These findings support the relevance of EEG measures for examining functional brain changes related to yoga practice.

Despite the growing body of literature on the neural effects of yoga practice, no systematic review to date has provided an integrated synthesis of EEG, MRI, and fMRI findings specifically focused on healthy participants. While previous reviews have addressed aspects of yoga-related neuroimaging, they have either focused on single modalities or included mixed clinical and non-clinical populations, thereby limiting the specificity of conclusions regarding yoga-related neuroplasticity in healthy brains (Desai et al., 2015; van Aalst et al., 2020). The present systematic review aims to address this gap by providing an updated and focused synthesis of neurophysiological and neuroimaging evidence up to 2025, with particular emphasis on functional connectivity and brain network organization in healthy individuals.

## 2 Methods

### 2.1 Search strategy

The research question (“What structural and functional changes occur in healthy brains with yoga practice?”) was formulated following the Population, Intervention, Comparison, Outcome (PICO) strategy.

The searches for the articles used for this review were performed on Web of Science, PubMed and PsycINFO on February 9, 2025, with the words and Boolean terms: “yoga” AND (“functional connectivity” OR “structural activity” OR “brain structure” OR “brain function”). There were no time range nor specifications set to the search, obtaining 123 results on Web of Science, 59 results on PubMed and 34 results on PsycINFO, for a total of 216 articles.

This systematic literature review was conducted following the Preferred Reporting Items for Systematic Reviews and Meta-Analyses (PRISMA) guidelines, which establish a series of criteria to follow to assure the quality of the review (Liberati et al., 2009; Moher et al., 2009).

The restriction to English-language publications reflects standard practice in systematic reviews, as English is the predominant language of peer-reviewed journals with high methodological standards. Importantly, the included studies were conducted across diverse geographical and cultural contexts, including non-Western countries such as India—the country of origin of yoga—thereby supporting the cultural breadth of the evidence synthesized in this review.

### 2.2 Study selection

The articles chosen for this review were screened twice: the first screening was of the title and abstract, and the second screening was of the Methods section. The selection criteria for the screening were as follows:

Inclusion criteria were (a) empirical published studies, (b) published in English, Spanish, or Dutch, (c) included healthy participants, (d) included either an experienced yoga practitioner group and a novice comparison group, and/or a pre-evaluation and post-evaluation of a yoga training group.

Exclusion criteria were (a) systematic reviews, meta-analyses, case studies, book chapters, dissertations, and protocols, (a) studies including participants with cognitive impairment or clinical disorders, (c) studies without a comparison group and without pre–post evaluation, (d) Studies including insufficient yoga exposure (e.g., only basic instructions without formal training), (e) studies reporting only peripheral physiological measures without neuroimaging outcomes.

**Figure 3:**
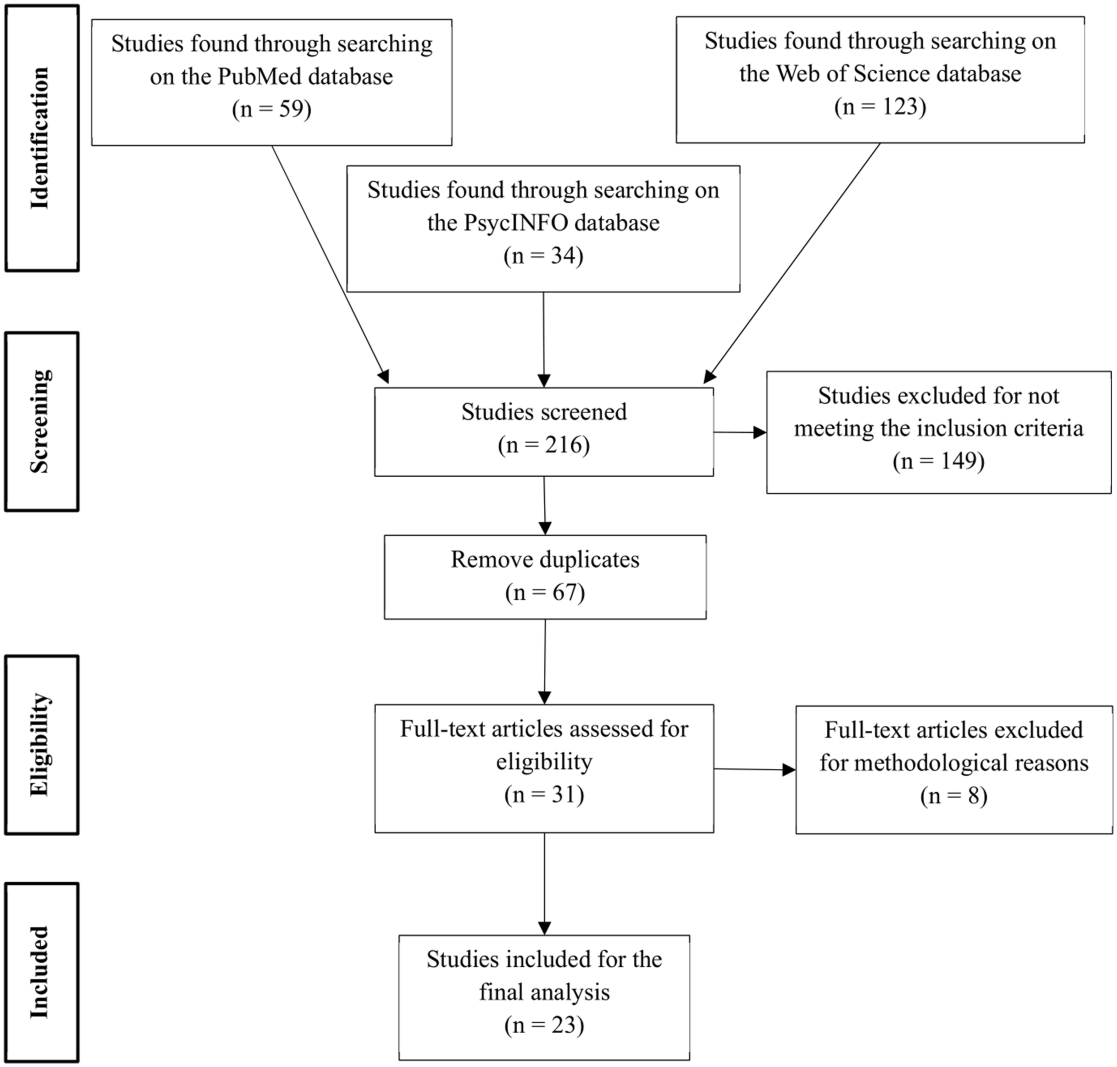
PRISMA flow diagram of the study selection process.

As shown in Figure 3, of the original 216 results, 149 were excluded. Of the remaining 67, 36 were duplicates, leaving 31 for the second screening. The second screening consisted of reading the full articles, with special attention to the Methods section to identify variables related to the research question: number of participants, their sex, age, and experience with yoga; the procedure followed, the measuring instruments and the structure/function studied. Having done this, one study was excluded as participants were novices and received no formal yoga training (only basic instructions) before the measurements were made; a second study was excluded due to the selection of novice participants with only two yoga sessions accompanied by transcranial direct current stimulation (tDCS), a neuro-stimulation technique that modulates cortical excitability; one study was excluded for only having salivary cortisol level measurements; one study was excluded for being a machine learning accuracy test; one study was excluded for not providing sample characteristics of the healthy participant group, only collective sample characteristics of a mixed group of healthy and unhealthy participants; and three more were excluded for having only one measure (no pre–post) of one group (without a comparison group).

No restrictions were applied regarding participants’ age range, the minimum duration or the intensity of yoga practice, or the year of publication, in order to provide a comprehensive synthesis of the available neuroimaging evidence of yoga.

During this process, some study validity criteria were taken into account, such as the attrition bias in the studies, which was not higher than 10% in any case. Another aspect was how participants were selected and assigned to the experimental or control group: all studies selected participants based on availability and, in some cases, a certain amount of experience with yoga, whilst the control groups (if there were any) were matched in age, sex, education, and other variables, with no experience. And, finally, in cases of doubt as to maintaining or discarding certain studies, experts in the field were consulted.

Figure 3 summarises the screening and selection procedure, whilst Table 1 presents the final 23 articles’ titles, first authors, year of publication, journals, countries, number of citations according to Google Scholar in March 2025, and a code for each publication. These 23 articles were first categorised based on if they were functional studies, structural studies, or both; following which they were analysed to determine their methodological aspects, such as their sample characteristics, types of yoga, imaging techniques, and study design (use of a pre-post evaluation, use of a comparison group, yoga training duration, etc.). This methodological information, along with a summary of each study’s results, is reflected in the Appendix.

**Table 1:** Articles included in the systematic review.

Code	First author and year	Journal	Cites	Country
1	Barrós-Loscertales et al. (2021)	Frontiers in Human Neuroscience	23	Spain
2	Dodich et al. (2019)	Brain and Behavior	52	Italy
3	Fialoke et al. (2024)	Scientific Reports	2	India
4	Fingelkurts et al. (2016b)	Self and Identity	33	Finland
5	Fingelkurts et al. (2016a)	Cognitive Processing	75	Finland
6	Gard et al. (2014a,b)	Frontiers in Aging Neuroscience	161	United States
7	Gard et al. (2015)	Frontiers in Human Neuroscience	96	United States
8	Gothe et al. (2018)	Frontiers in Integrative Neuroscience	85	United States
9	Gotink et al. (2018)	Brain Imaging and Behavior	69	Netherlands
10	Hernández et al. (2016)	PLoS ONE	134	Spain
11	Hernández et al. (2018)	Neuroscience	80	Spain
12	Malipeddi et al. (2024)	Mindfulness	2	India
13	Martínez et al. (2020)	Neuroscience	94	Argentina
14	Novaes et al. (2020)	Frontiers in Psychiatry	127	Brazil
15	Pérez-Diaz et al. (2024)	PLoS ONE	2	Spain
16	Santaella et al. (2019)	Frontiers in Aging Neuroscience	42	Brazil
17	Shrivastava et al. (2023)	Cureus	3	India
18	Simon et al. (2017)	Journal of Cognitive Enhancement	17	Sweden
19	Singleton et al. (2021)	Brain Sciences	12	United States
20	Thomas et al. (2014)	Frontiers in Human Neuroscience	46	Australia
21	Tymofiyeva et al. (2021)	NeuroImage: Clinical	25	United States
22	Villemure et al. (2015)	Frontiers in Human Neuroscience	169	Canada
23	Vishnubhotla et al. (2021)	Frontiers in Psychology	23	United States

The methodological quality and risk of bias of the included studies were formally assessed using validated appraisal tools appropriate to each study design. Of the 23 studies included in this review, one randomized controlled trial was evaluated using the Cochrane Risk of Bias 2 (RoB 2) tool (Sterne et al., 2019), while the remaining 22 non-randomized studies were assessed using the ROBINS-I tool (Sterne et al., 2016). The results of this assessment are summarized in Table 2.

**Table 2:** Methodological quality and risk of bias assessment of the included studies.

Code	Study design	Quality assessment tool	Main sources of bias	Overall risk of bias
1	Cross-sectional (experts vs. controls)	ROBINS-I	Confounding, participant self-selection	Moderate risk
2	Non-randomized intervention study	ROBINS-I	Confounding, lack of randomization, self-reported outcomes	Moderate risk
3	Pre–post design without randomization	ROBINS-I	Confounding, participant selection	Moderate risk
4	Cross-sectional (experts vs. controls)	ROBINS-I	Confounding due to prior experience	Moderate risk
5	Pre–post design without randomized control	ROBINS-I	Confounding, absence of active control	Moderate risk
6	Cross-sectional (experts vs. controls)	ROBINS-I	Confounding (age, cognitive activity)	Moderate risk
7	Cross-sectional (experts vs. controls)	ROBINS-I	Confounding, participant selection	Moderate risk
8	Cross-sectional study	ROBINS-I	Confounding, self-selection	Moderate risk
9	Population-based observational study	ROBINS-I	Residual confounding	Moderate risk
10	Cross-sectional (experts vs. controls)	ROBINS-I	Lifestyle-related confounding	Moderate risk
11	Cross-sectional + meditation state comparison	ROBINS-I	Confounding, participant selection	Moderate risk
12	Cross-sectional (experts, novices, controls)	ROBINS-I	Confounding by level of experience	Moderate risk
13	Cross-sectional study	ROBINS-I	Confounding, participant selection	Moderate risk
14	Randomized controlled trial	RoB 2	Lack of blinding, self-reported outcomes	Some concerns
15	Cross-sectional (experts vs. controls)	ROBINS-I	Confounding, participant selection	Moderate risk
16	Cross-sectional (older adults)	ROBINS-I	Healthy aging-related confounding	Moderate risk
17	Cross-sectional (experts, novices, controls)	ROBINS-I	High confounding	Moderate risk
18	Non-randomized pilot study	ROBINS-I	Selection bias, lack of preregistration	Moderate risk
19	Cross-sectional (experts vs. controls)	ROBINS-I	Psychosocial confounding	Moderate risk
20	Cross-sectional (experts vs. novices)	ROBINS-I	Confounding, participant selection	Moderate risk
21	Non-randomized longitudinal intervention	ROBINS-I	Confounding, mixed outcome measures	Moderate risk
22	Cross-sectional study	ROBINS-I	Confounding, participant selection	Moderate risk
23	Intensive non-randomized intervention	ROBINS-I	Confounding, participant selection	Moderate risk

Overall, most non-randomized studies were judged to present a moderate risk of bias, primarily due to confounding factors and participant self-selection into yoga practice. Importantly, bias related to outcome measurement was generally low, as most studies relied on objective neuroimaging techniques such as MRI, fMRI, or EEG. The single randomized controlled trial showed some concerns of bias, mainly related to the lack of participant blinding and the use of self-reported psychological outcomes.

## 3 Results

Given the heterogeneity of the included studies, the interpretation of the reported neural effects required consideration of participant characteristics and practice-related variables. As detailed in the Appendix, findings varied according to participants’ level of experience, duration and intensity of yoga practice, age, and the specific type of yoga examined. Studies involving long-term or highly experienced practitioners—often with several years or decades of regular practice—more consistently reported structural and functional brain differences, including changes in default mode network (DMN) organization, increased grey matter volume in regions such as the insula and hippocampus, and greater network integration.

In contrast, intervention and pre–post studies involving novice participants or short-term training programs, ranging from a few days to several weeks, more frequently reported state-dependent or training-related effects. These included reductions in anxiety and negative affect, decreased amygdala reactivity, and transient modulation of DMN activity. Participant age also appeared to influence the pattern of findings, with studies in older adults emphasizing network efficiency, resilience, and preservation of cognitive function. By comparison, studies with younger or mixed-age samples more often reported changes related to emotional regulation, attentional control, and self-referential processing. Finally, the type of yoga practiced differed substantially across studies, with more meditative traditions being more strongly associated with DMN modulation, while more physically oriented practices were more often linked to hippocampal and sensorimotor-related changes.

Of the 23 articles, three studied structural changes through MRI, 15 studied functional connectivity changes (six EEG, nine fMRI), and five studied both types of changes with MRI and fMRI. Regarding the sample characteristics, most studies had predominantly female samples, with only six studies that had more men than women. The average ages ranged between 16.4 and 66.5 years, and experience with yoga varied between none prior to the study training and a maximum experience of 30 years. Sahaja Yoga Meditation (SYM) was the most studied type of yoga (five studies), followed by investigations that studied multiple types of yoga (four studies).

Regarding functional neuroimaging paradigms, the included studies were differentiated based on whether they employed resting-state conditions or other acquisition paradigms. Resting-state paradigms were used in studies examining spontaneous brain activity or functional connectivity in the absence of an explicit task (Barrós-Loscertales et al., 2021; Fingelkurts et al., 2016b; Fingelkurts et al., 2016a; Gard et al., 2014a,b; Gard et al., 2015; Martínez et al., 2020; Santaella et al., 2019; Thomas et al., 2014; Villemure et al., 2015). In contrast, the remaining studies employed task-based paradigms, meditation- or practice-induced states, baseline-plus-meditation designs, or structural neuroimaging approaches, and therefore do not constitute resting-state paradigms (Dodich et al., 2019; Fialoke et al., 2024; Gothe et al., 2018; Gotink et al., 2018; Hernández et al., 2016; Hernández et al., 2018; Malipeddi et al., 2024; Novaes et al., 2020; Pérez-Diaz et al., 2024; Shrivastava et al., 2023; Simon et al., 2017; Singleton et al., 2021; Tymofiyeva et al., 2021; Vishnubhotla et al., 2021).

### 3.1 Structural changes

**Figure 4:**
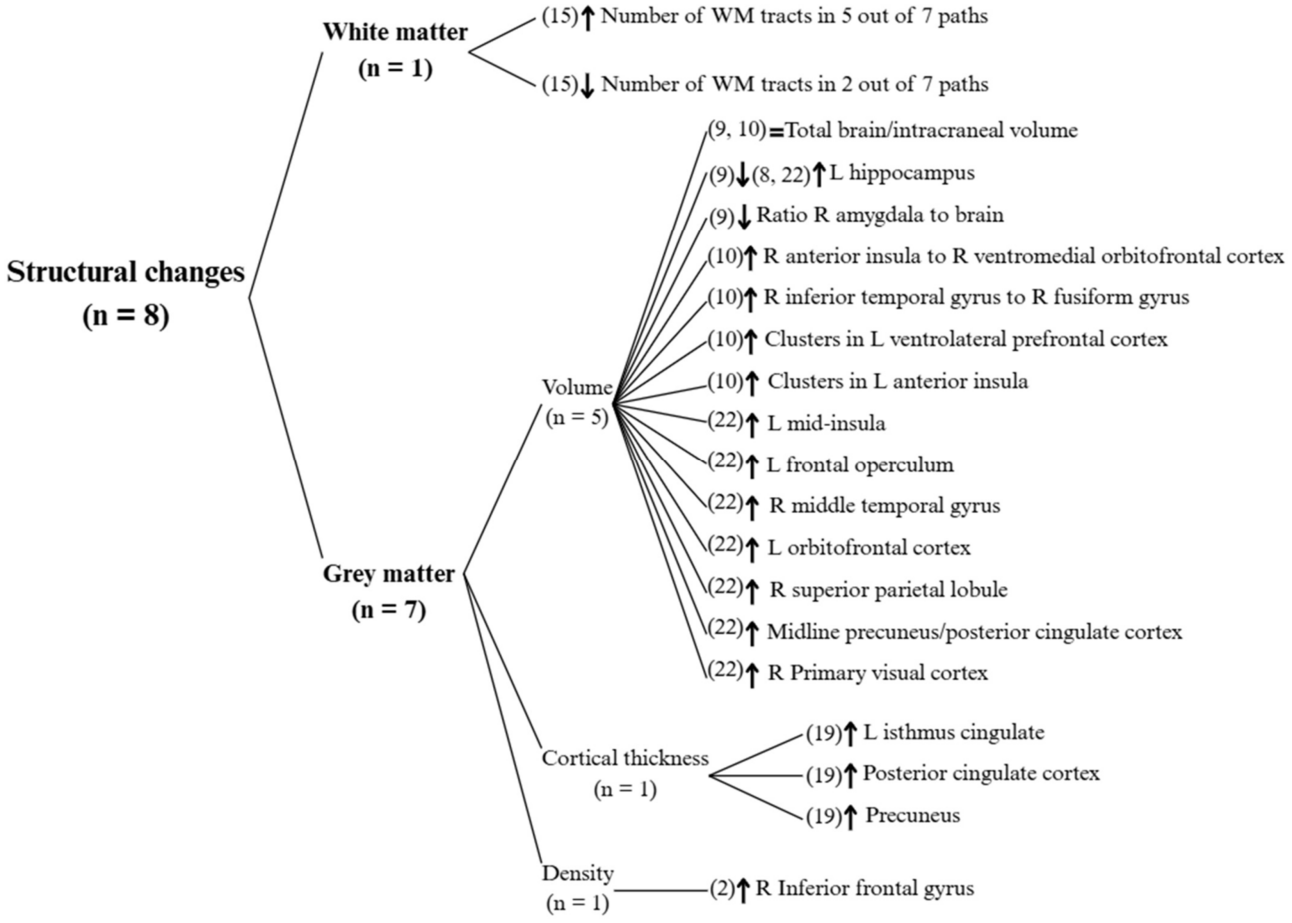
Structural changes in the human brain with yoga practice. L, left; R, right; WM, white matter. Only statistically significant results are shown.

In total, eight studies analysed structural changes in the human brain with yoga practice, which can be divided into WM changes and GM changes, summarised in Figure 4.

#### 3.1.1 White matter changes

Only one study (Pérez-Diaz et al., 2024) focused on the white matter (WM) changes, noting that in five of the seven WM paths analysed, people with experience in yoga had a higher number of tracts, such as between both amygdalae and the left anterior cingulate cortex (ACC). In the remaining two paths, yoga practitioners had a lower number of tracts than inexperienced participants, including in the left anterior insula (AI).

#### 3.1.2 Grey matter changes

Of the seven studies that analysed GM changes, two found that the total intracranial volume did not change between yoga practitioners and non-practitioners (Gotink et al., 2018; Hernández et al., 2016), despite there being some GM volume changes in certain areas, such as a decrease in right amygdala GM volume (Gotink et al., 2018), and an increase in GM volume stretching from the right AI to the right ventromedial orbitofrontal cortex (vmOFC), which correlated positively with depth of mental silence and daily frequency of thoughtless awareness (Hernández et al., 2016).

Other GM volume changes are varied among the studies. For example, two studies found an increase in volume for the insula, one in left AI clusters (Hernández et al., 2016) and the other in the left mid-insula (Villemure et al., 2015) both correlating positively with amount of experience. This latter study also found increases in the left frontal operculum, the right middle temporal gyrus (both of which correlated positively with experience), the right primary visual cortex, the posterior cingulate cortex (PCC), and the right superior parietal lobule (all three of which had a positive correlation with weekly practice).

There were contradictory results regarding the GM volume of the left hippocampus in yoga practitioners, with two studies finding an increase in volume that correlated with amount of practice (Gothe et al., 2018; Villemure et al., 2015), whilst one other found a decrease in volume with no significant correlations (Gotink et al., 2018).

One study analysed cortical thickness in yoga practitioners (Singleton et al., 2021), finding increases in the left isthmus cingulate, the precuneus and the PCC, all of which presented a positive correlation with total weighted scores for ego development in the Maturity Assessment Profile (MAP). Another study (Dodich et al., 2019) found an increase in GM density in the right inferior frontal gyrus, correlating with increased self-reported well-being.

### 3.2 Functional connectivity changes

A total of 20 studies analysed functional connectivity (FC) changes in the human brain with yoga practice, through EEG measurements and fMRI measurements.

#### 3.2.1 Electroencephalogram results

**Figure 5:**
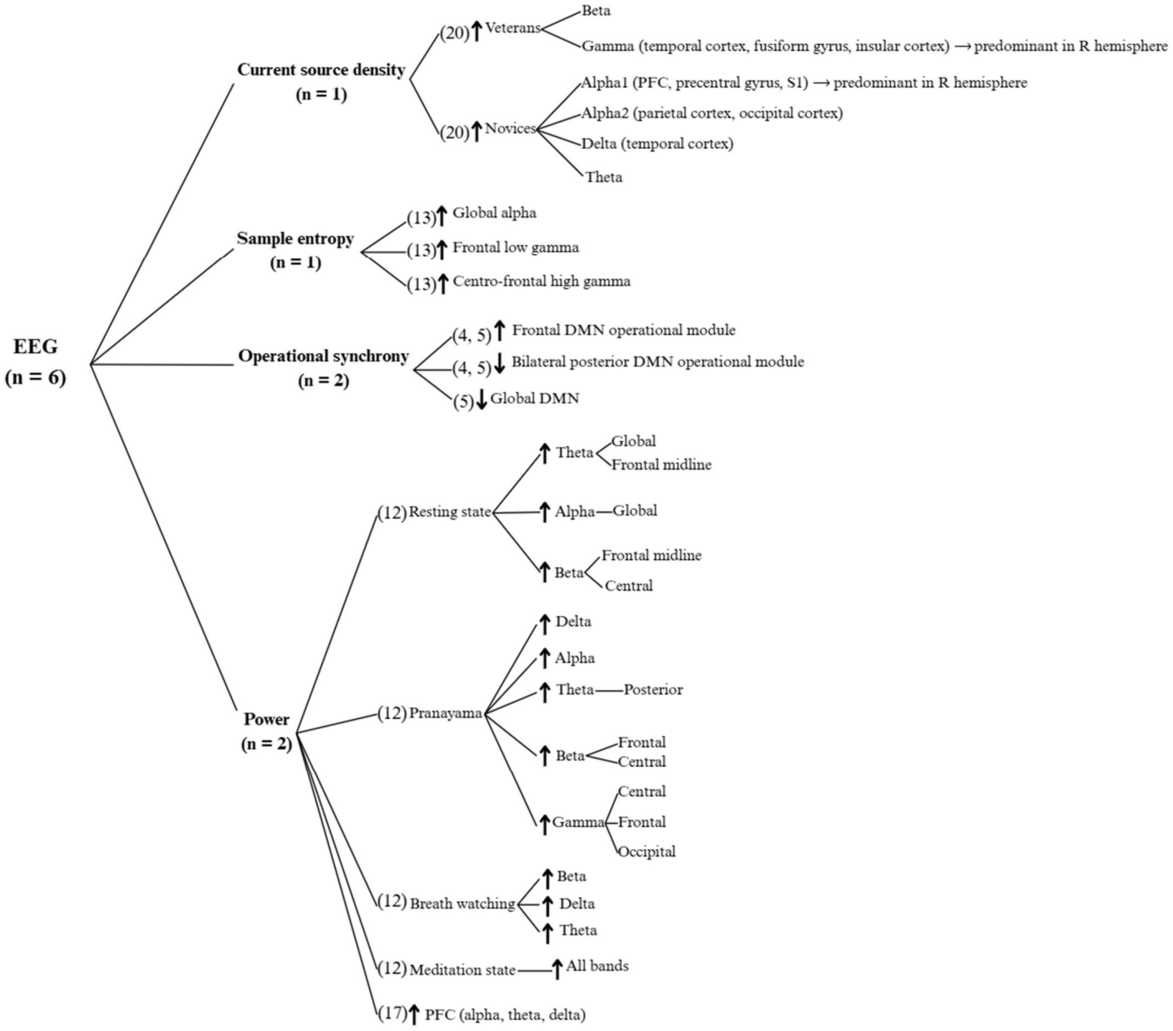
EEG results of functional connectivity changes in the human brain with yoga practice. DMN, default mode network; PFC, prefrontal cortex; R, right; S1, primary somatosensory cortex. Only statistically significant results are shown.

As shown in Figure 5, six studies used EEG as their imaging technique. Two studies (Fingelkurts et al., 2016b; Fingelkurts et al., 2016a) analysed changes in operational synchrony (OS), with one finding a decrease in global Default Mode Network (DMN) OS (Fingelkurts et al., 2016a), and both finding an increase in OS in the frontal DMN operational module and a decrease in the bilateral posterior DMN operational module. These changes in DMN activity correlated with self-reported calmness, happiness, self-agency, simpler subjective experiences and slower thought speed.

Another two investigations studied changes in frequency band power (Malipeddi et al., 2024; Shrivastava et al., 2023). Shrivastava et al. (2023) reported an increase in alpha, delta and theta band power in the prefrontal cortex (PFC) in all states. Malipeddi et al. (2024) found larger increases in the yoga group compared to the control group in different bands for different areas () depending on the state the participants were in. Specifically, in the meditation state, experienced practitioners had no change in band power, whilst novices and non-practitioners had reduced band power. These band power results correlated positively with self-reported well-being, meditation depth, and non-attachment in practitioners, whilst controls reported higher drowsiness, stress, mental distress and mind-wandering.

Of the remaining two EEG studies (Martínez et al., 2020; Thomas et al., 2014), one focused on sample entropy (SE) changes (Martínez et al., 2020), finding increases in global alpha, frontal low gamma and centro-frontal high gamma bands in a meditation group, and an increase in high gamma in a Hatha Yoga group. The second study (Thomas et al., 2014) focused on current source density (CSD), finding increases in beta and gamma bands in experienced yoga practitioners compared to novice practitioners; and increases in alpha1, alpha2, delta and theta bands in novice yoga practitioners, compared to experienced practitioners. Novice practitioners reported higher meditation depth than experienced practitioners, with the biggest difference during the mantra condition, which is when alpha1 activity was highest in novices.

#### 3.2.2 Functional magnetic resonance imaging results

**Figure 6:**
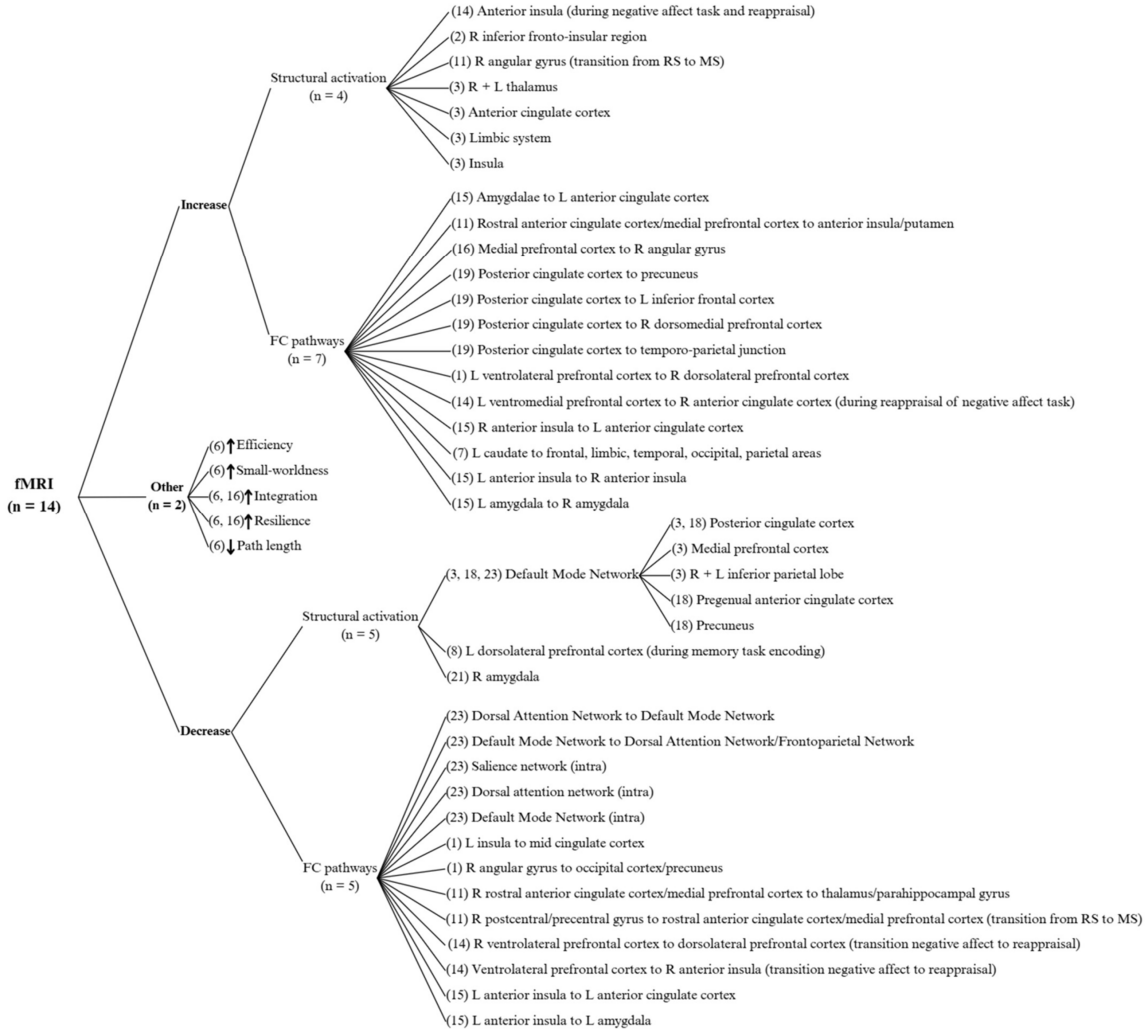
fMRI results of functional connectivity changes in the human brain with yoga practice. R, right; L, left; RS, resting state; MS, meditation state; FC, functional connectivity. Only statistically significant results are shown.

As summarised in Figure 6, there were 14 fMRI studies included in this review. Three studies (Fialoke et al., 2024; Simon et al., 2017; Vishnubhotla et al., 2021) found a decrease in DMN structural activity, specifically in the PCC (Fialoke et al., 2024; Simon et al., 2017), the pregenual ACC, the precuneus, the medial prefrontal cortex (mPFC) and both inferior parietal lobes. One study (Fialoke et al., 2024) found no DMN deactivation despite an increase in auditory and motor region activity. Another study (Tymofiyeva et al., 2021) found a decrease in right amygdala activation. All other structural activation and FC results are varied, with no coincidences nor contradictions between studies.

Regarding decreases in connectivity, one study (Vishnubhotla et al., 2021) found a reduction in FC pathways within the DMN, the dorsal attention network (DAN) and the salience network (SN); and other studies found FC decreases from the right angular gyrus to the left occipital cortex (Barrós-Loscertales et al., 2021), the left insula to the mid cingulate cortex (Barrós-Loscertales et al., 2021), and the rostral ACC to the thalamus (Hernández et al., 2018), among others.

Increases in structural activity were found in the insula, the right inferior fronto-insular region and the right angular gyrus. Some of the FC pathways with increases include: both amygdalae to the left ACC, the left ventrolateral prefrontal cortex (vlPFC) to the right dorsolateral prefrontal cortex (dlPFC), the mPFC to the right angular gyrus, the left insula to the right insula, the left amygdala to the right amygdala, the PCC to the precuneus, the PCC to the left inferior frontal cortex, the PCC to the right dorsomedial prefrontal cortex (dmPFC), and the PCC to the temporoparietal junction (TPJ).

These studies also investigated the psychological effects of those changes through different questionnaires and tasks. Five studies (Dodich et al., 2019; Fialoke et al., 2024; Gotink et al., 2018; Hernández et al., 2018; Novaes et al., 2020) found improved emotional regulation, through changes in structural activity and FC in and between regions such as the ACC, PCC, PFC, amygdalae, precuneus and insular and limbic regions; four studies (Barrós-Loscertales et al., 2021; Dodich et al., 2019; Malipeddi et al., 2024; Novaes et al., 2020) reported improved executive/cognitive control, through changes in structural activity and FC in and between regions such as the PFC, fronto-insular regions, and insular-cingulate pathways; three studies (Barrós-Loscertales et al., 2021; Fialoke et al., 2024; Hernández et al., 2018) found decreased mind-wandering through changes in structural activity and FC in and between regions such as the mPFC and the ventral attention network; three studies (Barrós-Loscertales et al., 2021; Dodich et al., 2019; Hernández et al., 2018) found an improvement in attention control through changes in structural activity and FC in and between regions such as the dorsal attention network and ventral attention network, and cingulo-insular and cingulo-amygdalar regions.

Two studies (Fialoke et al., 2024; Simon et al., 2017) found reduced self-referential processing through changes in structural activity and FC in and between regions such as the PCC and precuneus; one study (Hernández et al., 2018) reported increased mental silence through cluster activation in the ACC and the mPFC; one study (Gothe et al., 2018) found increased working memory efficiency through a reduced need to recruit the dlPFC for cognitive processing; and one study (Dodich et al., 2019) reported increased well-being, alongside changes in cognitive control and attentional allocation. There were contradictory results regarding stress levels, with one study (Gotink et al., 2018) finding that yoga practitioners had higher baseline self-reported stress levels than the average population, and another study (Malipeddi et al., 2024) finding that yoga practice reduces self-reported stress levels.

Other results include an increase in network integration and resilience (Gard et al., 2014a,b; Santaella et al., 2019), as well as an increase in small-worldness and efficiency, and a decrease in path length.

## 4 Discussion

The aim of this systematic review was to gain insight into the current literature regarding the effect that yoga has on brain structure and brain function in healthy individuals.

To facilitate the integration and comparison of findings across studies, a cross-study synthesis table was constructed (Table 3). This table summarizes the main neural effects reported across neuroimaging modalities, highlighting consistent and divergent patterns in relation to brain regions, networks, and associated psychological domains. Across studies, the most recurrent findings include modulation of default mode network activity and connectivity, alterations in limbic structures—particularly reduced amygdala reactivity and variable hippocampal structural changes. In addition, both functional and structural involvement of prefrontal, cingulate, and insular regions linked to executive control, emotional regulation, and interoceptive awareness were consistently reported (Gard et al., 2014b; Villemure et al., 2015; Gothe et al., 2018; van Aalst et al., 2020). The table also illustrates that these effects vary systematically according to participant experience, study design, and type of yoga practice, with long-term practitioners showing more trait-like structural and network-level adaptations, and short-term or intervention studies more frequently reporting state-dependent functional changes (Fingelkurts et al., 2016a; Fingelkurts et al., 2016b; Dodich et al., 2019; Santaella et al., 2019). By organizing results across these dimensions, the table provides an integrative overview. It complements the narrative synthesis, supports interpretation within existing theoretical frameworks, and underscores the methodological heterogeneity characterizing the current literature.

**Table 3:** Cross-study neuroimaging patterns associated with yoga practice.

Imaging modality	Convergent brain regions/networks	Recurring pattern across studies	Code	Evidence
EEG	Frontal and fronto-central regions; posterior DMN-related sites	Increased alpha and theta activity; gamma modulation mainly reported in experienced practitioners	Studies 3, 6, 9, 14	Patterns vary according to yoga style, practitioner expertise, and EEG metric (power, synchrony, entropy)
fMRI (resting-state)	Default mode network (DMN); prefrontal cortex (PFC); anterior cingulate cortex (ACC); insula–limbic connectivity	Altered functional connectivity, often interpreted as reduced DMN dominance and enhanced regulatory control	Studies 2, 5, 8, 11, 17	Direction and strength of effects depend on preprocessing pipelines and seed-based vs. network-level analyses
fMRI (task/meditation)	PFC, ACC, insula, hippocampus, and amygdala	State-dependent modulation of activation and connectivity during attention or emotion regulation tasks	Studies 1, 4, 10, 15	Strongly influenced by task paradigm and baseline condition
Structural MRI	PFC/ACC; hippocampus; insula; temporal–parietal regions	Differences in gray matter volume, cortical thickness, or density in regions related to self-regulation and emotional processing	Studies 7, 12, 16, 18, 21	Evidence is predominantly cross-sectional; longitudinal findings remain limited

Overall, standardized effect sizes reported across the included studies were predominantly large, with fewer moderate effects and only a small number of small effects (Table 4). Large effects were more frequently observed in intervention studies and in samples of experienced practitioners, whereas moderate effects were mainly associated with correlational analyses. Notably, several studies did not report effect sizes, particularly those employing voxel-wise neuroimaging analyses or machine-learning approaches. This lack of reporting limits quantitative comparability across findings and underscores the importance of improved reporting consistency in future studies.

**Table 4:** Standardized effect sizes reported across included studies.

Code	Relationship/outcome assessed	Effect size(s) (*r*, *R*^2^, *η*^2^)	Interpretation
1	Cognitive control (Simon task) and FC–behavior association	*r* = 0.34 (Simon task); *r* = −0.46 (insula–cingulate FC ↔ interference)	Moderate to moderate–large
2	Training-related structural changes (group × time) and well-being–brain associations	*η*^2^*p* ≈ 0.38 (GM changes); *r* = 0.62–0.72 (well-being ↔ brain measures)	Large to very large
3	Meditation experience ↔ DMN functional connectivity during Yoga Nidra	*r* = −0.31 to −0.35	Moderate
4	DMN operational synchrony (frontal ↑, posterior ↓)	*r* ≈ 0.52 (estimated, Wilcoxon)	Large
5	Pre–post changes in EEG functional connectivity within the DMN	*p* < 0.05–0.001	Significant changes
6	Group differences in cognition and functional network organization	*η*^2^p = 0.17–0.24 (network metrics); *r* = 0.29–0.37 (mindfulness ↔ networks/cognition)	Moderate to large
7	Caudate functional connectivity (degree centrality)	*r* = 0.45–0.73 (derived from *t*)	Moderate to very large
8	Differences in left hippocampal gray matter volume and task-related activation between yoga practitioners and controls	*t*(24) = −2.57, *p* = 0.017; *d* = −0.85	Large
9	Association between meditation/yoga practice and right amygdala volume, including longitudinal change	*β* = −31.8 mm^3^ (*p* = 0.005); *β* = −24.4 mm^3^ (*p* = 0.031)	Significant associations
10	Differences in whole-brain and regional gray matter volume between long-term meditators and controls	*F* = 10.45 (*p* = 0.002)	Significant statistics
11	Mental silence ↔ rACC functional connectivity	*r* = 0.80–0.83 (positive FC); *r* = −0.67 (negative FC)	Large to very large
12	Isha Yoga practice (novice vs. advanced vs. controls) → EEG oscillatory power	Cluster-level *t*-values ≈ 3.0–5.0, *p* < 0.05	Robust EEG differences
13	Group differences in EEG entropy and post-hoc comparisons	*η*^2^ = 0.41–0.44 (entropy); *r* = 0.38–0.51 (post-hoc)	Moderate–large to large
14	Clinical and neurofunctional changes following pranayama	*r* = 0.55–0.72 (clinical change ↔ brain measures)	Large
15	Frequency of mental silence ↔ ACC–amygdala connectivity	*r* ≈ −0.44	Moderate
16	Group differences in resting-state functional connectivity within the DMN	*p* < 0.05 (FWE-corrected)	Significant evidence of changes in functional connectivity
17	Discrimination between Rajayoga meditators with different levels of experience	Accuracy = 84.7%, sensitivity = 83.3%	High classification performance
18	Differences in prefrontal EEG functional connectivity and network organization across meditation states and practitioner groups	*p* < 0.05–0.001	Significant network changes
19	DMN suppression following mantra meditation training	*η*^2^*p* ≈ 0.75–0.82	Very large
20	Psychosocial development and DMN connectivity	*η*^2^*p* = 0.53 (development); *r* ≈ 0.50 (DMN ↔ development)	Large to very large
21	Group differences in EEG source activity between intermediate and advanced Satyananda Yoga	Voxel-wise *F* statistics ≈ 1.9–2.0, *p* < 0.05	Statistically significant
22	Anxiety levels and amygdala connectivity	*r* = 0.65	Large
23	Meditation experience/practice ↔ gray matter volume	*r* = 0.52–0.91; *R*^2^ = 0.54–0.81	Large to very large

Some studies did not report standardized effect sizes but provided statistical comparison measures (e.g., *t*/*F* statistics, corrected *p*-values, or classification metrics) indicating significant group or state differences without quantifying effect magnitude.

The findings of this systematic review can be understood within existing theoretical models proposing that yoga influences brain function through the interaction of top-down cognitive control mechanisms and bottom-up physiological regulation processes (Taylor et al., 2010; Gard et al., 2014a,b). Across neuroimaging modalities, convergent patterns emerged in brain regions and networks involved in executive control, emotional regulation, interoceptive awareness, and self-referential processing, including prefrontal, cingulate, insular, limbic, and default mode network regions (Gard et al., 2014b; Villemure et al., 2015; Gothe et al., 2018).

These patterns are broadly consistent with the executive homeostatic network proposed by Taylor et al. (2010), which emphasizes the integrative role of the PFC, ACC, and insula in regulating cognitive, emotional, and physiological processes. Similarly, the observed modulation of attentional networks, DMN dynamics, and autonomic-related regions aligns with the self-regulatory framework described by Gard et al. (2014a,b), which highlights coordinated regulation across attentional, emotional, and physiological domains. However, given the heterogeneity, the findings reviewed here should be interpreted as compatible with these theoretical frameworks rather than as direct empirical tests of the models. Despite substantial variability across studies, several convergent findings were identified. Specifically, three studies consistently reported reduced structural DMN activity in yoga practitioners compared to non-practitioners; two studies found decreased right amygdala activity; two studies reported increased operational synchrony in the frontal DMN alongside decreased synchrony in bilateral posterior DMN regions; and two studies observed increased gray matter volume in the left hippocampus (Fingelkurts et al., 2016a; Fingelkurts et al., 2016b; Santaella et al., 2019; Gotink et al., 2018; Villemure et al., 2015). In contrast, one study reported a decrease in hippocampal gray matter volume, highlighting the presence of divergent structural findings (Gotink et al., 2018).

Most other functional and structural results neither converged nor directly contradicted one another, which is likely attributable to multiple methodological factors. These include differences in sample characteristics (e.g., size, age, and level of yoga experience), study designs (e.g., intervention versus observational, pre–post versus cross-sectional, presence or absence of control groups), heterogeneity in yoga styles, and the lack of consensus regarding the definition of yoga and its distinction from meditation (Cramer et al., 2016a; Schmalzl et al., 2015; van Aalst et al., 2020). Additionally, technical challenges associated with acquiring neuroimaging data during *asana* practice further constrain the direct assessment of certain yoga components (Desai et al., 2015).

Regarding limbic and hippocampal structural findings, an apparent contradiction emerges across studies. Some report increased hippocampal volume, whereas others report reduced hippocampal and amygdala volume. This discrepancy may be partly explained by differences in study design and practice operationalization. The study reporting increased hippocampal volume was based on a small case–control design with a relatively homogeneous sample of yoga practitioners and conceptualized yoga as an active mind–body practice, likely emphasizing postural and movement-based components (Villemure et al., 2015). These components engage memory, spatial navigation, and sensorimotor integration processes, which are closely linked to hippocampal structure (Villemure et al., 2015; Rivest-Gadbois and Boudrias, 2019). In contrast, the findings of reduced amygdala and hippocampal volume were derived from a large population-based cohort study that grouped meditation, yoga, and breathing practices under a single exposure variable without distinguishing between contemplative and physically active techniques (Gotink et al., 2018). Such heterogeneity may reflect the inclusion of practices targeting emotional regulation and stress reduction. These processes are more closely associated with amygdala plasticity (Kinser et al., 2012; Schmalzl et al., 2015).

Similarly, inconsistencies in DMN-related findings appear to be strongly influenced by methodological differences. Reduced DMN connectivity is primarily observed during meditation-based practices, reflecting state-dependent effects, whereas resting-state studies in experienced practitioners more often report functional reorganization of DMN subcomponents rather than uniform suppression (Simon et al., 2017; Fingelkurts et al., 2016a; Santaella et al., 2019). These differences are closely related to the distinction between state and trait effects, as well as to participants’ level of experience (Fingelkurts et al., 2016b; Gard et al., 2014a).

Additional variability arises from differences in the operational definition of yoga and meditation. Some studies focus predominantly on contemplative techniques, while others include broader yoga practices integrating movement and breathing components, which may engage distinct neural processes (Schmalzl et al., 2015; Cramer et al., 2016a). Furthermore, lifestyle changes commonly associated with long-term practice—such as increased physical activity, enhanced stress awareness, or altered coping strategies—are rarely controlled for in neuroimaging studies. These factors may influence brain network organization, particularly DMN dynamics, especially in population-based designs (Villemure et al., 2015; van Aalst et al., 2020).

Notably, studies reporting higher perceived stress among yoga or meditation practitioners correspond to large population-based observational designs, whereas reductions in stress are primarily reported in intervention or pre–post studies assessing changes over time (Clarke et al., 2015; Zhang et al., 2021; Kinser et al., 2012). Beyond differences in study design, this apparent discrepancy may be explained by participant self-selection, as individuals experiencing higher baseline stress may be more likely to initiate yoga or meditation practices as coping strategies (Cramer et al., 2016b; Nourollahimoghadam et al., 2021). Differences in stress measurement—ranging from single-item self-reports to validated psychometric instruments—may further contribute to divergent findings (Birdee et al., 2015; Kinser et al., 2012). Finally, lifestyle factors associated with yoga and meditation practice are seldom controlled for and may independently influence reported stress levels (Villemure et al., 2015; van Aalst et al., 2020).

The structural findings reported by Pérez-Diaz et al. (2024) may be particularly relevant in light of the established role of amygdala–anterior cingulate interactions in emotional processing and regulatory control. The ACC has been described as a central node within executive and affective regulatory networks (Taylor et al., 2010; Gard et al., 2014a,b), and its bidirectional connections with the amygdala are considered important for top-down modulation of emotional responses. Thus, increased white matter tracts between these regions in experienced practitioners could reflect enhanced structural integration within limbic–prefrontal pathways associated with emotion regulation. Conversely, reduced tract numbers involving the anterior insula may be associated with differential engagement of interoceptive and salience-processing networks, which are also implicated in self-regulatory mechanisms (Lou et al., 2011; Schmalzl et al., 2015). The contradictory findings regarding left hippocampal volume are noteworthy. Gothe et al. (2018) and Villemure et al. (2015) reported increases associated with practice, whereas Gotink et al. (2018) reported decreases without significant correlations. These discrepancies are particularly relevant given the established role of the hippocampus in memory processes, stress regulation, and neuroplasticity (Taylor et al., 2010; Chen, 2024). The positive associations with practice may reflect experience-dependent structural plasticity, whereas inconsistencies across studies could be explained by methodological differences, including sample size and study design, as well as variability in stress levels or practice characteristics.

Overall, the findings of the present review are broadly consistent with self-regulatory models of yoga practice, particularly the framework proposed by Gard et al. (2014a,b). Across studies, yoga-related changes in emotional regulation, cognitive control, and attentional processes were most consistently associated with structural and functional connectivity alterations in regions such as the insula, prefrontal cortex, and cingulate cortex (Gard et al., 2014b; Gothe et al., 2018; Santaella et al., 2019). These regions form part of large-scale networks supporting attention, self-monitoring, and top-down regulation. However, discrepancies remain regarding the specific subregions involved, with some studies linking emotional regulation to anterior cingulate connectivity and others emphasizing posterior cingulate or default mode network involvement (Hernández et al., 2018; Simon et al., 2017). In addition, accumulating evidence suggests that different components of yoga may exert distinct neurobiological effects, as postural practice, breathing exercises, and meditation engage partially overlapping but functionally specific neural systems (Villemure et al., 2015; Schmalzl et al., 2015). Despite this variability, the overall pattern of findings supports the interpretation of yoga-related neural effects within a distributed self-regulatory framework (Gard et al., 2014a; Villemure et al., 2015).

Another review focused on brain waves and structural activation (Desai et al., 2015), finding that brain wave activity increased, especially in the frontal cortex, which is consistent with the EEG findings in the current review and further supports the involvement of prefrontal regions in yoga practice.

Taken together, these findings have both theoretical and practical implications. The recurrent structural and functional involvement of prefrontal, cingulate, insular, hippocampal, and default mode regions suggests that yoga, as a multimodal mind–body practice, may be associated with experience-dependent neuroplastic adaptations within distributed brain networks supporting self-regulation, interoception, and cognitive–emotional integration. Rather than reflecting isolated regional effects, the overall pattern is consistent with models proposing dynamic interactions between top-down executive systems and bottom-up limbic and interoceptive pathways, thereby aligning with contemporary models of large-scale brain network organization in contemplative practices. At the same time, the heterogeneity observed across studies highlights the need for more research, and clearer operational definitions of yoga interventions, systematic differentiation of postural, breathing, and meditative components, consistent reporting of standardized effect sizes. Future research should prioritize adequately powered longitudinal and controlled neuroimaging designs to clarify the magnitude, specificity, and temporal trajectory of yoga-related neural changes. The selection of articles reviewed leads to believe that, despite the possible limitations, there is sufficient evidence to recommend the practice of yoga for stress awareness, stress management, and emotional regulation.

The primary limitation of this review is the substantial methodological heterogeneity across the included studies, both in terms of the neuroimaging techniques employed and the methodological approaches adopted. The studies vary widely with respect to imaging modality (EEG, structural MRI, and fMRI), experimental design (cross-sectional, pre–post, and intervention studies), analytic strategies, and outcome measures. This variability precludes a straightforward and unified synthesis of the literature and makes it difficult to draw clear, overarching conclusions. Such heterogeneity, while reflecting the current state of the field, limits direct comparability across studies. Additional limitations include the time constraints inherent to conducting a review of this scope within the context of an academic thesis. These constraints restricted the extent of additional analyses that could be performed. Finally, study selection was not conducted entirely through a peer-based consensus process, raising the possibility that some relevant studies may have been inadvertently excluded.

The main conclusion of this review is that there are inconsistent results regarding the impact of yoga on brain structure and function in healthy individuals due to methodological differences, thus, the main recommendations for future studies around the neuropsychological effects of yoga are to clarify the definition of yoga and to establish a more homogenous methodology between studies of the same kind of neuroimaging technique (i.e., similar methodologies for all MRI studies, similar methodologies for all fMRI studies, etc.). Another recommendation is to differentiate between the different components of yoga, as Villemure et al. (2015) indicate that postures, breathing exercises and meditation have different effects.

## Data Availability

The original contributions presented in the study are included in the article/Supplementary Figures, further inquiries can be directed to the corresponding author.
