# Supplementary material for: How yoga shapes the brain: a systematic review
Source: Front Neurosci. 2026 Apr 13;20:1718808. doi: 10.3389/fnins.2026.1718808 (PMC13111208; doi:10.3389/fnins.2026.1718808)
Supplement: Supplementary file 1 [file Table_1.docx]

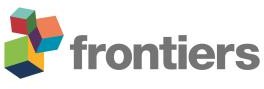


Appendix

Appendix. Summary of the reviewed articles’ methodological characteristics and results.

| Code | n (♀) | X̅ age | Functional or structural | Imaging technique | Type of yoga | Training | Experience | Comparison group | Effect | Other |
| --- | --- | --- | --- | --- | --- | --- | --- | --- | --- | --- |
| 1 | 23  (17) | 46.5 | FC | fMRI | SYM | No | 14.1 years avg (84.7min/day) | =, no EXP: 23 (17), 46.9yrs | E > CONT:   - ↑FC L vlPFC - R dlPFC [cognitive control, conflict resolution, attention networks -ventral and dorsal-] - ↓FC L insula [interoceptive perception, saliency and DMN] - mid cingulate cortex [anterior DMN node, conflict detection] [cognitive control, conflict resolution] - ↓FC R angular gyrus [ventral attention]/precuneus [posterior DMN node] - occipital cortex/precuneus   Not sig FC AI - inferior temporal gyrus  Possible collineality with no FC dif. when GM vol regressed out. Simon task interference correlated with ↓FC L insula - mid cingulate cortex  SYM improves dorsal+ventral attention, executive control (inhibitory self-control); - correlation ventral attention-DMN (no mind-wandering/irrelevant thinking; associated with better mental health);  better performance in cognitive control tasks with conflict detection and attention control | Go-no go and Simon task |

| 2 | 12 (2) | 21.63 | FC + S | MRI, fMRI | SYM | pre-post, 4 weeks (16h) training, experienced instructor: 15min theory, 45min meditation | No | 30 (12),  22.16yrs | E:   - ↑ well-being, ↓ fatigue and ↓ dissatisfaction - ↑GM density R inferior frontal gyrus [well- being] - ↑FC R inferior fronto-insular region (executive control)   Capacity to consciously modulate internal and external attention control mechanisms. Beneficial effects of SYM on the efficiency of cognitive control and attentional allocation | TCI-56 short version, ad-hoc well-being and emotionality |
| --- | --- | --- | --- | --- | --- | --- | --- | --- | --- | --- |
| 3 | 28 (4) | 27.57 | FC | fMRI | YN | 2-3/week during 1 month prior to eval. | 2516.98h avg | =, no EXP  (21.50h avg):  24 (4),  25.96yrs | Auditory and motor regions activation, but NO DMN deactivation  E:   - ACC, insula, limbic [emotional regulation] activation. Bilat thalamic activation - ↓FC in 4 DMN seeds (PCC, mPFC, R IPL, L IPL)   Not sig:   - Dif E-CONT in RS pre-post YN - E decoupling in DMN connectivity - CONT ↑ coupling of DMN   ↓activation mPFC 🡪 ↓self-related thinking/mind- wandering  Correlation: total duration of meditation practice and ↓FC of 4 DMN seeds |  |

| 4 | 5 (3) | 53 | FC | EEG | Multiple | No | 3.9yrs avg | No EXP, 10  (6), 51.7yrs, | E:   - Simpler subjective experiences, slower thought speed, ↑ experience of self-agent, ↑ calmness and ↑ happiness - Mild ↑OS EEG frontal DMN OM [sense of self] - ↓OS EEG bilat posterior DMN OM [R: interoceptive and exteroceptive bodily sensory processing; L: oneself, narrative thoughts, inner speech, reinterpretation of   events] | Self-report demographics and practice |
| --- | --- | --- | --- | --- | --- | --- | --- | --- | --- | --- |
| 5 | 10 (6) | 51.7 | FC | EEG | Kriya Yoga | pre-post eval. 4 months training (20min daily). Pre: 1 week before training. Post: 1 week after training | No | No | ↓OS EEG general DMN  ↓OS bilat posterior OM [continuity of "I"]  ↑OS frontal OM [sense of self/agency] |  |
| 6 | Y: 16  (69%)  M: 16  (63%) | Y: 49.38  M: 54.06 | FC | MRI, fMRI | Kripalu Yoga, VIP | No | Y: 13534h M: 7458h | =, no EXP: 15 (60%),  52.93yrs | Y>M>CONT  Y: ↓ decline of fluid intelligence, shorter characteristic path length, ↑ global efficiency, ↑ small-worldness, ↑ network integration, ↑ network resilience  M: ↓ decline of fluid intelligence, shorter characteristic path length, slight ↑ global efficiency, slight ↑ small-worldness, ↑ network integration, moderate ↑ network resilience  Mindfulness 🡪 ↑ fluid intelligence, ↑ integration and ↑ resilience in RS networks | MMSE,  Raven's APM, AMNART,  FFMQ, self- report of physical and cognitive exercise |

| 7 | 13  (31%) | 38.15 | FC | MRI, fMRI | VIP | No | 4831h | =, no EXP: 16 (44%), 36yrs | Y: ↑ degree centrality (bilat) not correlated with age or fluid intelligence, ↑FC  M: ↑ degree centrality (bilat), ↑FC  CONT: less degree centrality (bilat), weaker connectivity.  Fig 3 indicates stronger connectivity of caudate (especially L) with frontal, limbic, temporal, occipital and parietal areas (less with insular, cerebellar and basal ganglia regions) | Raven's APM, self-report demographics and practice |
| --- | --- | --- | --- | --- | --- | --- | --- | --- | --- | --- |
| 8 | 13  (12) | 35.8 | FC + S | MRI, fMRI | Multiple (mostly Hatha) | No | 9.31yrs (4/week) | =, no EXP: 13 (12), 35.7 | ↑ L hippocampal vol No dif:   - Vol: R hippocampus, thalamus, caudate - Shape: L hippocampus, R hippocampus, thalamus, caudate   L dlPFC ↓activation during encoding (not maintenance or retrieval) | 6min walk test, psychosocial and demographic questionnaires, Sternberg Working Memory Task |
| 9 | 289  (73%) | 61.9 | S | MRI | Multiple (mostly meditation) | Technically no, but longitudinal comparison with data 5yrs prior (slightly different sample) | Over 10yrs | Less than 1h practice per week: 3453  (53%), 64.3 | Higher stress (Y and breathing) and DEP (M) in E, no dif for ANX  Vol (E=Y+M+breathing):   - No dif total brain vol E-CONT - E < CONT: ratio R amyg-total brain vol - E < CONT: L hippocampus Not sig vol: - Y>breathing>CONT: amyg - E < CONT: R amyg - E > CONT: L amyg | CES-D,  HADS-A, self- report stress levels (1-5) |

|  |  |  |  |  |  |  |  |  | DEP score correlation with R amyg vol in CONT, NOT in E  Longitudinal results = ↓ R amyg vol [neg emotion and immediate action taking], no dif in L amyg or hippocampus vol |
| --- | --- | --- | --- | --- | --- | --- | --- | --- | --- |
| 10 | 23  (17) | 46.5 | S | MRI | SYM | No | 14.1 years avg (84.7min/day) | =, no EXP: 23 (17), 46.9yrs | TIV no dif M > CONT:   - GM vol, especially R AI-R vmOFC and R inferior temporal gyrus-R fusiform gyrus - Vol clusters in L vlPFC and L AI No CONT > M for GM vol |
| 11 | 23  (17) | 46.5 | FC + S | MRI, fMRI | SYM | No | 14.1 years avg (84.7min/day) | =, no EXP: 23 (17), 46.9yrs | M: + correlation depth of mental silence and daily frequency of thoughtless awareness (also with age and experience)  MS: + correlation depth of mental silence with cluster in rostral ACC-medial PFC. M > CONT:   - GM vol (not sig) in rostral ACC-medial PFC - Bilat: ↑FC rACC/mPFC-AI/putamen, and MS   > RS   - R: ↓FC rACC/mPFC-   thalamus/parahippocampal gyrus, and MS = RS  RS:   - No dif FC rACC-mPFC |

|  |  |  |  |  |  |  |  |  | - Bilat: No correlation between depth of mental silence and rACC/mPFC-AI/putamen - R: - correlation depth and rACC/mPFC- thalamus/parahippocampal gyrus   RS 🡪 MS: ↑FC R angular gyrus, and ↓FC R postcentral/precentral gyrus (with rACC/mPFC)  Downregulation of sensory processes and mind wandering with ↓FC regions. Meditative state maintained by ↑FC areas. rACC/mPFC role as hub node in top-down emotion and attention control |  |
| --- | --- | --- | --- | --- | --- | --- | --- | --- | --- | --- |
| 12 | E: 42  (18)  N: 33  (14) | E = 35.57  N = 31.66 | FC | EEG | SNY | No | E = 5507.80h N = 1637.24h | =, no EXP: 28 (16), 31.14 | E>N>CONT: wellbeing, nonattachment, meditation depth  CONT>N>E: drowsiness, stress, mental distress RS:   - E > CONT frontal midline theta - CONT > E centro-occipital gamma   [relaxation, mental quiescence, blissful states; enhanced internalized attention and relaxation]   - E > N in global alpha and theta power and fronto-midline and central beta power [tonic alertness] - CONT > N in L posterior regions alpha and centro-occipital gamma [CONT ↑alpha 🡪 mindwandering]   Pranayama: | Self-report demographics and practice |

- CONT: ↑delta power (anterior) and theta power (frontal midline)
- E+N: ↑power on all freq bands [beta and gamma sense of wakefulness and perceptual clarity]
- No dif E and N (E localized vs. N global, inverted U-shape due to effortlessness in E]
- N > CONT in posterior theta power, frontal and central beta power, and gamma (frontal, central and occipital)
- E > CONT in gamma power (frontal and occipital)

Breath-watching:

- CONT ↑delta and theta (anterior), ↓ in alpha (occipital).
- N+E ↑ power in delta, theta and beta
- No dif between groups MS:
- CONT (all) and N (delta, beta, gamma): ↓ power
- E no dif
- E > CONT in all freq
- E > N in all freq except delta

|  |  |  |  |  |  |  |  |  | - N > CONT in all freq Pre-post: - E no dif. - N (localized) ↓ all freq - CONT (global) ↓ all freq Theta power 🡪 relaxation, attention,   metaawareness, bliss (all groups); alpha 🡪 relaxation and internalized attention; delta 🡪 relaxation; beta 🡪 cognitive control and tonic alertness; gamma 🡪 attention, moment to moment awareness and perceptual clarity (E) |  |
| --- | --- | --- | --- | --- | --- | --- | --- | --- | --- | --- |
| 13 | HT: 16 (2)  SNY: 16 (2)  VIP: 16 (2) | HT: 43  SNY: 40  VIP: 47 | FC | EEG | HT, SNY, VIP | No | Unknown | =, no EXP: 16  (5), 45yrs | HT: high gamma (unspecified location) SNY: not sig  VIP: ↑SE in alpha (global), low gamma (frontal) and high gamma (central-frontal) | Self-report demographics and practice |
| 14 | 15 (8) | 24 | FC | fMRI | Pranayama | 5 days guided training; 5/week during 4 weeks | No | =, no EXP: 15 (7), 26.2 | E > CONT: ↓ANX, ↓neg affect, ↑pos affect NEG:   - Interaction R amyg-bilat AI - E: ↑activity bilat AI - Neg affect: E least ↓activity in R amyg and AI REAP: ↑interaction L vmPFC-R ACC in E   NEG 🡪 REAP:   - ↑activity R AI | Perceived stress scale, PHQ-4, WHO- WBI, NAS-7, MEDEQ, SSS |

|  |  |  |  |  |  |  |  |  | - E > CONT: ↓FC R vlPFC-dlPFC and ↓FC bilat vlPFC-R AI. + correlation with ↓ANX  Lateral PFC regulation of affect and emotion. Cognitive reappraisal tasks recruit dlPFC and vlPFC (- correlation neg emotion) |  |
| --- | --- | --- | --- | --- | --- | --- | --- | --- | --- | --- |
| 15 | 20  (10) | 47 | FC + S | fMRI | SYM | No | 21.8yrs avg | =, no EXP: 20 (11), 46yrs | M > CONT:   - #WM tracts 5/7 paths - ↑FC R AI-L ACC - ↑FC L amyg-L ACC - ↑FC R amyg-L ACC - ↑FC L AI-R AI - ↑FC L amyg-R amyg CONT > M: - #WM tracts 2/7 paths - ↑FC L AI-L ACC - ↑FC L AI-L amyg |  |
| 16 | 20  (20) | 66.5 | FC | fMRI | Hatha Yoga | No | 15.1yrs avg | =, no EXP: 20 (20), 68.2 | RS: ↑FC mPFC-R AGr  Higher integration and resilience of networks, protect fluid intelligence |  |
| 17 | LTM: 13 (4)  STM: 11 (4) | LTM 32.54  STM 30 | FC | EEG | HM | No | LTM = 98.71  months avg (76.07min/day)  STM = 12.80  months avg (47.5min/day) | No EXP: 10  (3), 28.43yrs | ↑FC within PFC 🡪 LTM>STM>CONT in all conditions for r, PLV and CC. LTM<STM<CONT in all states for SP  r: delta, alpha [LTM]  PLV: delta, theta, alpha [LTM]  CC: delta, theta, alpha [LTM, STM] | MMSE, BDI, IADL |

|  |  |  |  |  |  |  |  |  | SP: delta, theta, alpha [LTM, STM] |  |
| --- | --- | --- | --- | --- | --- | --- | --- | --- | --- | --- |
| 18 | 8 (8) | 31 | FC | fMRI | Kundalini yoga | 3/week during 2 weeks (90min). Scan 1 week before,  after week 1,  after week 2 | No | No | Deactivation in DMN subregions (mantra vs finger tapping): precuneus, PACC, PCC  Training effect:   - Precuneus ↓activity both weeks - PACC ↓activity week 1 |  |
| 19 | Y: 16  (11)  M: 14  (9) | Y: 49.38;  M: 54.8 | FC + S | MRI, fMRI | Kripalu Yoga, VIP | No | Y: 13534h M: 7774h | =, no EXP: 16 (9), 52.5yrs | TWS MAP higher in M+Y vs. CONT, and M > Y  + correlation:   - TWS and cortical thickness in L isthmus cingulate, PCC and precuneus - TWS and FC PCC-precuneus and L IFC. - M and Y: TWS and FC PCC/precuneus-R dmPFC - Y: TWS and FC PCC/precuneus-TPJ - correlation CONT: - TWS and FC PCC/precuneus-R dmPFC - TWS and FC PCC/precuneus-TPJ | MMSE, MAP, AMNART, MSCEIT, SCS, FFMQ |
| 20 | 6 (3) | 54 | FC | EEG | Satyananda | No | 30yrs | 4 yrs exp avg: 6 (3), 42yrs | ↑Meditation depth through procedure, SYS > SYT SYS > SYT:   - CSD in theta and alpha1 all conditions - CSD in alpha2 for body-steadiness not mantra. | Self-report meditation depth |

|  |  |  |  |  |  |  |  |  | - Most higher CSD in R hemisphere alpha1, specifically precentral gyrus and primary somatosensory cortex - CSD voxels in R hemisphere delta (temporal cortex), alpha1 (PFC, precentral gyrus, primary somatosensory cortex), alpha2 (parietal cortex, occipital cortex)   SYT > SYS:   - CSD in beta and gamma - Most higher CSD in R hemisphere gamma. Body steadiness in fusiform gyrus, middle temporal gyrus, insula and anterior temporal lobe; mantra in insula and inferior prefrontal gyrus - CSD voxels in gamma (temporal cortex, fusiform gyrus, insular cortex)   Highest # sig dif in gamma and alpha1 |  |
| --- | --- | --- | --- | --- | --- | --- | --- | --- | --- | --- |
| 21 | 23  (13) | 16.4 | FC | fMRI | TARA | 12 weeks TARA  training, 90min, 1/week. 3 scans | No | No | ↓ ANX symptoms.  ↓R amyg node strength (1º scan > 2º scan).  + correlation symptoms and R amyg node strength Not sig, medium effect size:  - ↑ L caudate/L putamen node strength. | MASC,  RADS-2, self- report practice |

|  |  |  |  |  |  |  |  |  | - L caudate - correlation with ANX symptoms and somatic complaints; L putamen - correlation ANX and DEP symptoms - ↓ R caudate node strength   - + correlation R amyg node strength and anhedonia/neg affect  - + correlation L insula node strength and  anhedonia/neg affect |  |
| --- | --- | --- | --- | --- | --- | --- | --- | --- | --- | --- |
| 22 | 14 (9) | 37 | S | MRI | Multiple | No | 9.6yrs (8.6h/week) | 14 (9), 36.7yrs | CONT: - correlation GM and age. E: no correlation GM and age  + correlations:   - Exp and GM vol in L mid-insula, L frontal operculum, R middle temporal gyrus, L OFC - Weekly practice and GM vol in R SPL, L hippocampus, midline precuneus/PCC, R primary visual cortex   Postures > meditation predictor of hippocampal GM vol  #hours for posture predictor of SPL vol Postures and breath control best predict V1 vol Meditation best predictor of PCC vol | Self-report demographics and practice |
| 23 | 13 (5) | 34.9 | FC | fMRI | Samyama | 8 day program (CONT  received no instructions) | Yes (4 Isha programs completed) | No EXP: 4 (2),  62.8yrs | ↓FC within SN, within DAN and within DMN  ↓FC DAN-DMN |  |

↓FC DMN-DAN/FPN

+ correlation mindfulness and FC SN (SMG-ACC)

*Note.* “=”: matched; “<”: smaller/lower; “>”: larger/higher; “#”: number of; “+”/pos: positive; “-“/neg: negative; ACC: anterior cingulate

cortex; AGr: angular gyrus; AI: anterior insula; Amyg: amygdala; ANX: anxiety/anxious; CC: clustering coefficient; CONT: control group;

CSD: current source density; CSF: cerebrum spinal fluid; DAN: Dorsal Attention Network; DEP: depression/depressive; dif: difference;

DMN: Default Mode Network; E: experienced group; FC: functional connectivity; FPN: frontoparietal network; GM: grey matter; HM:

Heartfulness meditation; HT: Himalaya Yoga; IFC: inferior frontal cortex; L: left; LTM: long-term meditators; M: meditator group; MS:

meditation state; N: novice group; No Exp: no experience; OM: operational module; OS: operational synchrony; PACC: pregenual anterior

cingulate cortex; PCC: posterior cingulate cortex; PLV: phase-locking value; R: right; RS: resting state; S: structural; SE: sample entropy;

SN: salience network; SNY: Isha Yoga; SP: shortest path; SPL: superior parietal lobule STM: short-term meditators; SYM: Sahaja Yoga

Meditation; SYS: Satyananda student; SYT: Satyananda teacher; TARA: Training for Awareness, Resilience and Action; TIV: total

intracranial volume; TWS: total weighted score; VIP: Vipassana; WM: white matter; Y: yoga group; YN: Yoga Nidra.
